# The Extracellular Matrix Enriched With Exosomes for the Treatment on Pulmonary Fibrosis in Mice

**DOI:** 10.3389/fphar.2021.747223

**Published:** 2021-12-06

**Authors:** Yanzhen Yu, Xingzhi Liu, Zhe Zhao, Zhongjuan Xu, Yong Qiao, Yuanshuai Zhou, Hong Qiao, Junjie Zhong, Jianwu Dai, Guangli Suo

**Affiliations:** ^1^ School of Nano-Tech and Nano-Bionics, University of Science and Technology of China, Hefei, China; ^2^ CAS Key Laboratory of Nano-Bio Interface, Suzhou Institute of Nano-Tech and Nano-Bionics, Chinese Academy of Sciences, Suzhou, China; ^3^ Jiangsu Key Lab of Medical Optics, Suzhou Institute of Biomedical Engineering and Technology, Chinese Academy of Sciences, Suzhou, China; ^4^ Department of Molecular Biosciences, the University of Texas at Austin, Austin, TX, United States; ^5^ Department of Neurosurgery, Fudan University Huashan Hospital, National Key Laboratory of Medical Neurobiology, Institutes of Brain Science, Shanghai Medical College, Fudan University, Shanghai, China; ^6^ State Key Laboratory of Molecular, Developmental Biology, Institute of Genetics and Developmental Biology, Chinese Academy of Sciences, Beijing, China

**Keywords:** pulmonary fibrosis, extracellular matrix, miR-29, exosome, collagen-binding domain

## Abstract

Pulmonary fibrosis (PF) is a severe respiratory disease caused by lung microenvironment changes. TGF-β/Smad3 signaling pathway plays a critical role in the fibrotic process. MicroRNA-29 (miR-29) has proved to alleviate the occurrence of PF by downregulating TGF-β/Smad3 signaling pathway. The miRNA application encounters obstacles due to its low stability in body and no targeting to lesions. Exosomes can be used for therapeutic delivery of miRNA due to their favorable delivery properties. However, low efficiency of separation and production impedes the therapeutic application of exosomes. In this study, we developed a liquid natural extracellular matrix (ECM) enriched with miR-29-loaded exosomes for PF treatment. The collagen-binding domain (CBD)-fused Lamp2b (CBD-Lamp2b) and miR-29 were overexpressed in human foreskin fibroblast (HFF) host cells for the entrapment of miR-29-loaded exosomes in ECM of the cells. The repeated freeze-thaw method was performed to prepare the liquid ECM enriched with exosomes without destroying the exosomal membrane. In summary, this study developed a novel functional ECM biomaterial for therapy of PF, and also provided a promising gene therapy platform for different diseases by treatment with liquid ECM that is, enriched with exosomes loaded with different functional miRNAs.

## Introduction

Pulmonary fibrosis (PF) is a model fibrotic disease mainly caused by microenvironmental lung changes (Parker et al., 2014). Fibroblasts and myofibroblasts play essential roles in the pathological process *via* secreting growth factors, such as transforming growth factor (TGF-β), and excessive collagens (Yang et al., 2013). The inhibition of TGF-β1/Smad3 signaling has been reported to alleviate PF (Wang et al., 2016). For example, the TGF-β1/Smad3 signaling pathway was inhibited by microRNAs for the treatment of lung PF (Yang et al., 2013; Wang et al., 2016). Previous studies suggest that the microRNA-29 (miR-29) is one of the known TGF-β1-associated microRNAs involved in PF because it can regulate the expression levels of extracellular matrix (ECM) proteins such as collagen1a1 (Col1a1) and collagen3a1 (Col3a1) (Xiao et al., 2012; Montgomery et al., 2014). Although miR-29 has shown excellent advantages in treating PF, its clinical application faces some challenges, such as easy degradation by RNA enzyme, no targeting, and low stability *in vivo*. Recently, many powerful nano delivery systems have been developed for stable and efficient RNA delivery, such as viruses, liposomes, polymer nanoparticles, and exosomes (Paroo and Corey, 2004; Davis et al., 2009; Zhang et al., 2013; Li and Rana, 2014; Shu et al., 2014; Yang et al., 2014; Borna et al., 2015; Maghsoudnia et al., 2020). They have shown many advantages on low dose, high efficacy, stability and targeting.

Exosomes, nano-sized vesicles with diameters of 40–150 nm, are secreted by most eukaryotic cells and circulated in the extracellular environment (Wortzel et al., 2019; Duan et al., 2020; Miao et al., 2021). The exosomes play a functional role in cell-to-cell signaling interactions for the main contains the exosomes can deliver, including nucleic acids (RNA and DNA), proteins, lipids, and metabolites. When exosomes are uptaken by recipient cells, various biomolecules carried by exosomes are transferred to the recipient cells. This causes the response and even changes the functions of recipient cells (Wu et al., 2020). Exosomes have attracted extensive interest in the field of therapeutic applications due to their favorable properties for the delivery of RNAs (Lu and Huang, 2020). So far, exosomes-associated gene delivery has shown its therapeutic effects on brain liver diseases, cardiovascular diseases, and other diseases (Duan et al., 2020). But low efficiency of separation and production, difficulty in drug loading and targeting are common problems for therapeutic application of exosomes (Li P. et al., 2017; Lu and Huang, 2020; Zhang et al., 2020).

Extracellular matrix (ECM), a non-cellular three-dimensional macromolecular network secreted by cells and distributed surrounding the cells, plays a vital role in maintaining microenvironment for cells. Its main molecular complexes contain fibronectin, laminins, elastin, proteoglycans/glycosaminoglycans, collagens, and glycoproteins (Theocharis et al., 2016; Bhattacharjee et al., 2019; Zhang et al., 2019). ECM components form a complex network by binding each other as well as cell adhesion receptors. Cell surface receptors transduce signals into cells from ECM, which regulates diverse cellular functions such as survival, growth, migration, and differentiation. ECM has been applied as an ideal biomaterial to mimic cellular microenvironment for tissue engineering research and diseases treatment for its merits such as neutrality, good biocompatibility, degradability, and controllability. ECM was used to create a suitable bony microenvironment with cell-derived ECM and biodegradable β-tricalcium phosphate (β-TCP) (Kang et al., 2011). It was also used to form a dynamic microenvironment for stem cell niche (Gattazzo et al., 2014), to control cell culture (Barthes et al., 2015; Lin et al., 2020), and to maintain hematopoietic microenvironment (Klein, 1995). In our previous study, we developed a liquid natural ECM biomaterial enriched with insulin-degrading enzyme (IDE) and membrane metalloendopeptidase (MME) to reduce amyloid-beta (Aβ) peptide accumulation and Tau phosphorylation in Alzheimer’s disease cell models (Zhang et al., 2019). The ECM and exosomes are both synthesized and secreted by cells. Thereby, it is possible to modify the exosome membrane proteins to link exosomes to ECM by means of genetic engineering for the purpose of exosomes enrichment.

In this study, we developed a functional ECM biomaterial for PF treatment. We overexpressed collagen-binding domain (CBD)-fused Lamp2b (CBD-Lamp2b) and miR-29 in human foreskin fibroblast (HFF) cells, and further produced liquid ECM in which the exosomes loaded with miR-29 were remarkably enriched relying on the binding between CBD on exosomal membrane and collagen I in ECM. Our study demonstrated that this miR-29-loaded exosomes-enriched ECM (mi29-Exo-ECM) inhibited TGF-β1/Smad3 signaling pathway *in vitro*, and effectively alleviated the accumulation of PF *in vivo*. This study developed a type of novel functional ECM biomaterial that may hopefully facilitate PF therapy. In addition, a promising platform for gene therapy was developed to potentially treat different diseases with the liquid ECM that is, enriched with exosomes loaded with different functional miRNA.

## Materials and Methods

### Cell Culture

Human foreskin fibroblast (HFF) and human embryonic kidney cells (HEK 293T) were cultured in Dulbecco’s modified Eagle medium (DMEM) (GIBCO, ThermoFisher, Waltham, MA, United States) supplemented with 10% pre-selected fetal bovine serum (FBS) (GIBCO, ThermoFisher, Waltham, MA, United States), 1% penicillin and streptomycin (GIBCO, ThermoFisher, Waltham, MA, United States) in a 37°C humidified incubator with 5% CO_2_. Human umbilical cord mesenchymal stem cells (hUCMSCs) were cultured in Dulbecco’s modified Eagle’s medium/nutrient mixture F-12 HAM (1:1) (DMEM/F12, GIBCO, ThermoFisher, Waltham, MA, United States) containing 10% FBS and 1% penicillin/streptomycin in a 37°C humidified incubator with 5% CO_2_.

NIH 3T3 cells were maintained in DMEM with 10% FBS and kept in a 37°C incubator with a 5% CO_2_ air atmosphere, and TGF-β (Novoprotein, China) was added with 2 ng/ml. The total proteins of cells were harvested 24 h post-treatment.

### Plasmid Construction, Lentivirus Production, and Transduction

The cDNA encoding the lysosome-associated membrane protein-2b (Lamp2b) was cloned from HFF cells by polymerase chain reaction (PCR). The fused gene CBD-Lamp2b was generated by PCR using cDNA of Lamp2b as template. The DNA fragment of CBD-Lamp2b was subcloned into the reading frame of pLVX-IRES-Puro lentiviral vector (Clontech Laboratories, Mountain View, CA), and the new formed construct was named as pLVX-CBD-Lamp2b. MiR-29 oligos purchased from Genewiz (Jiangsu, China) were cloned into pLKO.1 (Addgene, United States) to form new construct named as pLKO.1-miR-29. All primers with restriction sites were listed in Supplementary Table S1.

For lentivirus production, HEK 293T cells were cultured to 70% confluence in 60 mm dishes and were co-transfected with packaging plasmids (pMD2.G and psPAX2) and lentiviral vectors (pLVX-CBD-Lamp2b) using lipofectamine 2000 (Invitrogen, Carlsbad, CA, United States). After 6 h of transduction, the medium was replaced with fresh medium. After 48 h of culture, the virus-containing supernatant was collected and filtered through a 0.22 μm pore size filter. The virus stocks were used to infect HFF cells with the presence of 8 μg/ml polybrene to form HFF-pLVX, HFF-pLVX-Lamp2b, HFF-pLVX-CBD-Lamp2b cell lines. After transfection, those cells were selected by 1 μg/ml of puromycin for 7 days to get the stable transfected cell lines. In order to overexpress miR-29 in exosomes, HFF-pLVX-CBD-Lamp2b cells were cultured to 80% confluence in 100 mm dish and co-transfected with pLKO.1-miR-29 using lipofectamine 2000. After 6 h of culture, the medium was replaced with fresh medium.

### Quantitative Real-Time Polymerase Chain Reaction

The qRT-PCR was performed according to previously reported method (Qiao et al., 2020) to confirm the overexpression of CBD-Lamp2b in HFF and the overexpression of miR-29 in exosomes from mi29-Exo-ECM. Briefly, total RNA was extracted from HFF-pLVX, HFF-pLVX-Lamp2b, HFF-pLVX-CBD-Lamp2b using TRIzol Reagent (Invitrogen, ThermoFisher, Waltham, MA, United States). The total RNA of exosomes was extracted with miRNeasy Mini Kit (Qiagen, Germany). The RNA was subsequently reverse transcribed to cDNA using High Capacity cDNA Reverse Transcription Kit (Thermo Fisher, Waltham, MA, United States), and miR-29 was reverse transcribed with specialized reverse primers (Supplementary Table S1). The qRT-PCR was performed with the Power SYBR Green PCR Master Mix (Applied Biosystems, TheomoFisher, Walltham, MA, United States) on 7,500 real time thermocycle instrument (Applied Biosystems, ThermoFisher, Walltham, MA, United States). The relative expression levels were expressed in arbitrary units (a.u.) while the Ct value of the gene of interest was normalized to that of β-actin or U6. The qRT-PCR primers for target genes (Lamp2b and miR-29) were designed using the Primer Express software (Version 2.0, Applied Biosystems, Walltham, MA, United States) and showed in Supplementary Table S1.

### Liquid ECM Material Preparation by Repeated Freeze-Thaw Method

The repeated freeze-thaw method was used for liquid ECM preparation (Lu et al., 2011, 2012; Xing et al., 2015; Fernández-Pérez and Ahearne, 2019; Li et al., 2019). Briefly, the cells were expanded to confluence in culture dish and continuously cultured for 14 days in DMEM containing 10% FBS, 50 μM ascorbic acid, and 1% penicillin/streptomycin in a 37°C humidified incubator with 5% CO_2_. After washing with phosphate-buffered saline (PBS) for three times, the cells were frozen in −80°C refrigerator for 2 h and then thawed in 37°C incubator for 5 min. This freeze-thaw process was repeated three times to rupture the cell membrane. Finally, the cell contents were removed by gently rinsing with PBS for three times. Subsequently, the decellularized ECM was dissolved in PBS and centrifuged at 10,000 g for 20 min at 4°C to sufficiently remove the cell membrane fragments; the supernatant was collected as the liquid ECM that was named as Exo-ECM. Western blot, transmission electron microscopy (TEM), scanning electron microscope (SEM) and nanoparticle tracking analysis (NTA) were performed to characterize the ECM biomaterials containing exosomes.

### Western Blot Analysis

Cells or exosomes were harvested and lysed in RIPA lysis buffer. The lysates were centrifuged at 12,000 g for 20 min at 4°C, and proteins in the supernatants were quantitated using the Lowry Protein Assay quantitation Kit (Beyotime, Shanghai, China). Lysates were fractionated on 10% SDS-PAGE gels and transferred to polyvinylidene difluoride (PVDF) membranes. After blocked with 5% bovine serum albumin (BSA) for 1.5 h, PVDF membranes were incubated with primary antibodies overnight at 4°C and HRP-conjugated secondary antibody for 2 h at room temperature. Finally, the membranes were visualized by chemiluminescence following standard protocols and photographed by Fujifilm LAS 4000 luminescent image analyzer (Fujifilm Life Science, Japan). The densities of protein bands were measured using ImageJ software. The antibodies against following protein were purchased from Cell Signaling Technology (Beverly, MA, United States): Smad3 (C67H9) Rabbit mAb, β-actin, GAPDH, Phospho-Smad3 (Ser423/425) (C25A9) Rabbit mAb, Alix (3A9) Mouse mAb, COL1A1 (E8I9Z) Rabbit mAb, α-Smooth Muscle Actin (D4K9N) XP^®^ Rabbit mAb, E-Cadherin (24E10) Rabbit mAb. The antibodies against following protein were purchased from Proteintech (United States): CD63 Polyclonal Antibody, HSP90 Monoclonal Antibody, LAMP2 Monoclonal Antibody, Collagen Type IV Polyclonal Antibody, Fibronectin Polyclonal Antibody, laminin, Aggrecan Polyclonal Antibody, Elastin Polyclonal Antibody, ATP1A1 Polyclonal Antibody, TGF Beta 1 Polyclonal Antibody. The antibody against TSG 101 was purchased from abcam (Cambridge, CB2 0AX, UK). The goat anti-rabbit or anti-mouse HRP conjugated secondary antibodies were purchased from Cell Signaling Technology (Beverly, MA, United States).

### Transmission Electron Microscopy and Scanning Electron Microscope

The scanning electron microscopy was used to observe and assess the entrapment of exosomes in ECM surrounding cells. The cells were expanded to confluence in glass slide and continuously cultured for 14 days in DMEM containing 10% FBS, 50 μM ascorbic acid, and 1% penicillin/streptomycin in a 37°C humidified incubator with 5% CO_2_. Then the cells were fixed in 2.5% glutaraldehyde in PBS (pH 7.4) overnight. After dehydration, critical point drying and spraying, the cells were analyzed with a scanning electron microscope (Hitachi SU8010, Japan). The morphological images of HFF cells were acquired, and the number of exosomes with diameter ranging from 40 to 150 nm surrounding cells was counted.

The transmission electron microscopy was used to observe the morphology of exosomes isolated from liquid ECM by ultracentrifugation. Exosomes were fixed in 2.5% glutaraldehyde in PBS (pH 7.4) overnight. Then the samples were rinsed in PBS and refixed in 1% osmium tetroxide at 4°C overnight. After dehydrated in increasing concentrations of acetone, samples were embedded in epoxy resin. Ultrathin sections were prepared and adsorbed to formvar-coated copper grids. Samples were stained with uranyl acetate and lead citrate and then photographed using Tecnai transmission electron microscope (FEI, United States).

### Nanoparticle Tracking Analysis

Exosomes were diluted with PBS to reach the recommended measurement range (10^6^ to 10^9^ particles/mL) and were analyzed using NanoSight NS300 instruments (Malvern Instruments, Inc., UK). The light scattered by the exosomes with laser illumination was captured by a camera and a video file of exosomes moving under Brownian motion was created. The NTA software tracked and analyzed particles individually from 10 to 2000 nm and the Strokes-Einstein equation was used to calculate their particle sizes together with an estimate of the concentration (Dragovic et al., 2011).

### Exosomal miRNA Analysis *via* Next-Generation Sequencing Technology

The next-generation sequencing technology was used to investigate the difference of RNAs content among the different types of exosomes from the media of HFF cells, HFF-Lamp2b cells and hUCMSCs, and from the ECM of HFF-CBD-Lamp2b cells. Small RNA sequencing was serviced by LC Sciences (Hangzhou, Zhejiang, China). The experimental process was performed under the standard steps provided by Illumina, including library preparation and sequencing experiments. Total RNA was extracted and purified using the mirVanaTM miRNA Isolation Kit without phenol (Ambion, United States). TruSeq Small RNA Sample Prep Kits (Illumina, San Diego, United States) were used to construct miRNA sequencing libraries. Total RNAs were ligated with the ligate 3′ and 5′ adapter using T4 RNA ligase (Epicentre, United States) at 28°C for 1 h. The adapter-ligated miRNAs were used as templates to perform PCR amplification and the cDNAs were purified with 6% TBE PAGE gels. The completed libraries’ quality and correctness were evaluated with qRT-PCR, and the high-throughput sequencing of the cDNA was performed on the HiSeq 2,500 (Illumina, United States). Raw reads were subjected to an in-house program ACGT101-miR (LC Sciences, Houston, Texas, United States) to remove adapter dimers, junk, low complexity, common RNA families (rRNA, tRNA, snRNA, snoRNA) and repeats. Then the clean reads were used for the analysis of small RNA data. The spearman’s correlation analysis, principal component analysis (PCA) and heat map analysis were performed to compare the miRNA differences in exosomes from each group.

### The Development of PF Model and the Treatment With ECM

C57BL/6 mice (∼25 g, male) were anesthetized by placing them in a chamber containing paper towels soaked with 40% isoflurane solution, and 2 mg/kg mouse of bleomycin (Selleck, United States) in 50 μL of 0.9% saline was administered intratracheally. To study the effect of materials on early fibrosis, we administered total 600 μg liquid ECM with about 25 pmol exosomes with or without miR-29 enrichment in one mouse at the 3rd day after bleomycin treatment and sacrificed the mice at the 14th day. The lungs were harvested for histological, immunohistochemical (IHC) and Western blot analysis.

### Histologic, Masson, and Immunohistochemical Analyses

Histologic, Masson and IHC analyses were performed according to the previously reported methods (Qiao et al., 2020) with modifications. For histological analysis, lung sections were stained with haematoxylin and eosin (H&E) and Masson. The antibodies against α-SMA (CST, Beverly, MA, United States), TGF-β (Proteintech, United States) were used for IHC staining in lung tissue sections, the percentage of TGF-β and α-SMA positive area (%) from IHC were quantified by ImageJ software. The stained sections were observed and the images were acquired using a microscope (ZEISS AXIO microscope, Axio Scope A1, United States).

### Quantify Degree of Fibrosis With Ashcroft Scoring System

The Ashcroft score was performed according to previously reported method (Ashcroft et al., 1988) to estimate severity of PF. Briefly, a paraffin section of lung stained by haematoxylin and eosin method, was systematically scanned in a microscope using a 10X objective. Each successive field was individually assessed for severity of interstitial fibrosis and allotted a score between 0 and 8 using a predetermined scale of severity. “0” represented normal lung; “1” and “2” represented minimal fibrous thickening of alveolar or bronchiolar walls; “3” and “4” represented moderate thickening of walls without obvious damage to lung architecture; “5” and “6” represented increased fibrosis with definite damage to lung structure and formation of fibrous bands or small fibrous masses; “7” represented severe distortion of structure and large fibrous areas; “8” represented total fibrous obliteration of the field. After examining the whole section the mean score of all the fields was taken as the fibrosis score for the section and was expressed correct to two decimal places.

### Hydroxyproline Assay

A HYP assay kit (Nanjing Jiancheng Bioengineering Institute) was used to measure the concentration of HYP in lung tissue samples. The experimental procedure was performed according the instruction. Briefly, the lung tissue (30 mg) was homogenized in 1 ml hydrolysate (95°C water bath for 20 min). After neutralizing the hydrolysates with NaOH, the mixture was diluted to 10 ml with water. Subsequently, the absorbance values of the prepared samples were detected at 550 nm. The results were expressed as μg hydroxyproline/mg wet lung.

### Statistical Analysis

All experiments were independently repeated 3 times and all measurements were done at least in triplicates. Statistical significance was determined by unpaired two-tailed student’s *t*-test (*p* < 0.05) and one-way ANOVA. *p* < 0.05 was considered statistically significant. All statistical analyses were performed using GraphPad Prism 5 (GraphPad Software, Inc., La Jolla, CA, United States).

## Results

**FIGURE 1:**
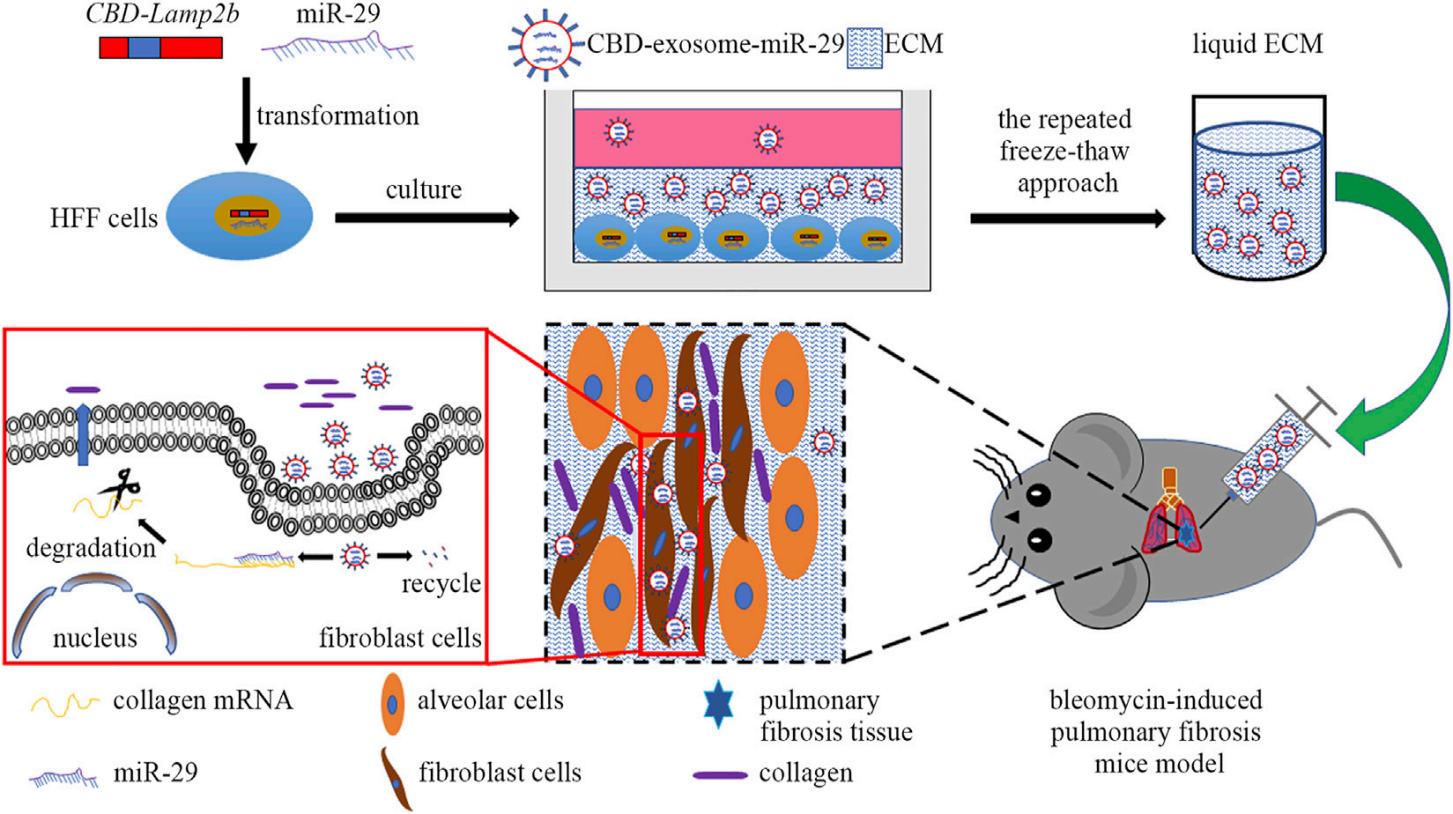
The scheme of this study includes four main steps: 1) construction of HFF cell lines that stably expressing CBD-Lamp2b and miR-29; 2) enrichment of CBD-exosomes in ECM; 3) production of liquid ECM; 4) treatment of pulmonary fibrosis mice with mi29-Exo-ECM biomaterial.

In this study, we attempted to develop a kind of functional biomaterial containing ECM microenvironment enriched with exosomes for repair of PF. The scheme of this study was shown in Figure 1. First, we overexpressed the fusion protein CBD-Lamp2b in HFF cells to entrap the secreted exosomes in ECM surrounding the cells by the binding of CBD on exosomal membrane with the collagen I in ECM. The expression cassette of the modified Lamp2b protein was shown in Supplementary Figure S1. Second, to obtain the liquid exosomes-enriched ECM without destroying the exosomal membrane, we performed repeated freeze-thaw method to remove cell contents and dissolve the decellularized ECM in PBS. Third, to endow the function of this biomaterial on PF therapy, we overexpressed miR-29 into the host HFF cells from which the miR-29-loaded exosomes (mi29-Exo) were generated. Finally, miR-29-loaded exosomes-enriched ECM (mi29-Exo-ECM) biomaterial was produced and applied for PF repair *in vivo*.

### Enrichment of Exosomes Surrounding the Host Cells

**FIGURE 2:**
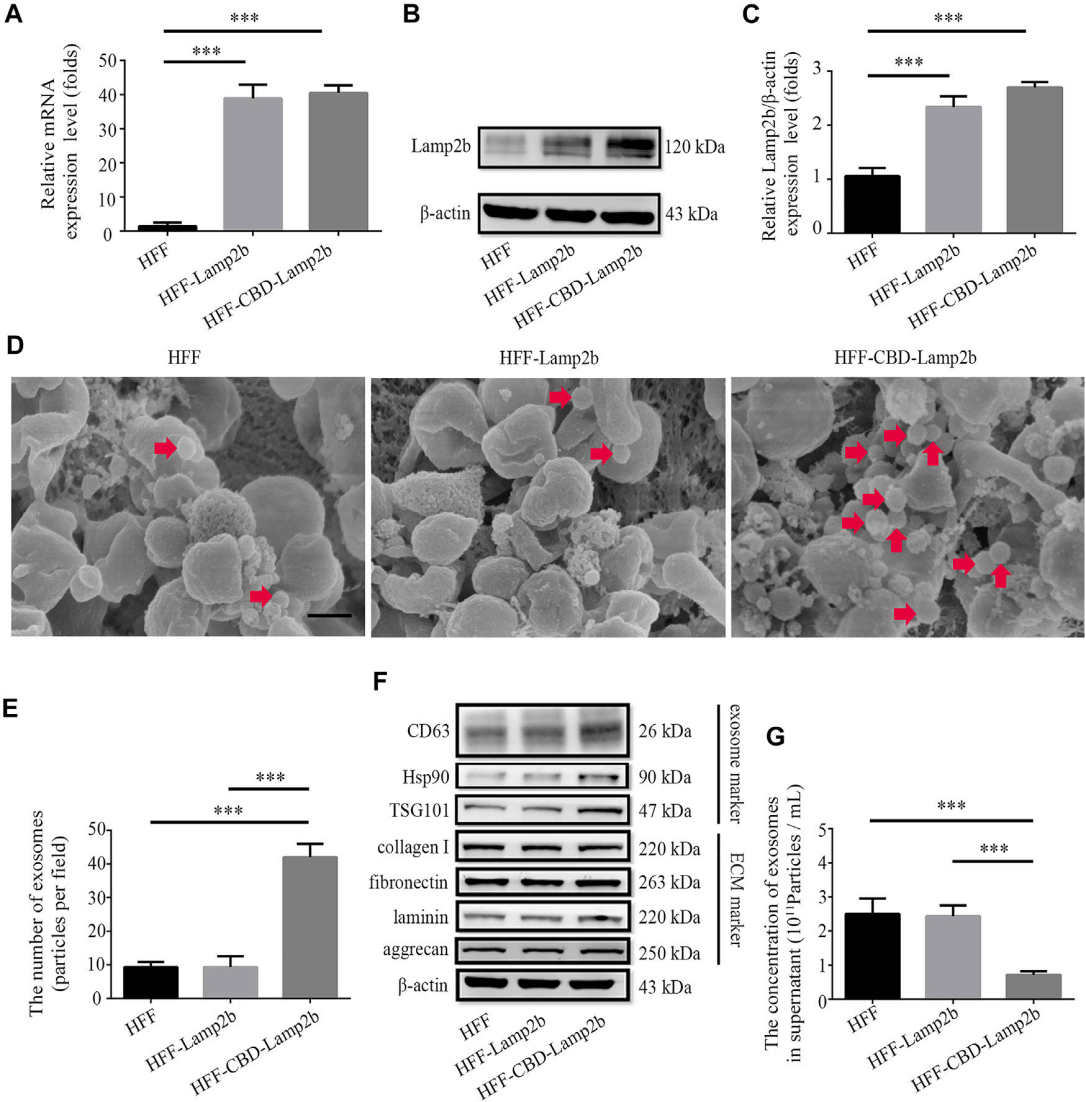
Enrichment of exosomes surrounding the host cells. Overexpression of Lamp2b in HFF cells determined by **(A)** qRT-PCR and **(B)** Western blot. **(C)** Relative expression level of Lamp2b was quantified. The β-actin was used as an internal control. **(D)** Representative SEM images of indicated cell surface. The red arrows indicate the exosomes surrounding the host cells. Scale bar = 250 nm. **(E)** The number of exosomes in the view. **(F)** Western blot analysis of exosomal markers and ECM markers in indicated cells. **(G)** The NTA analysis of exosomal number in cell culture supernatants. Data are represented as mean ± SD (*n* = 3). ANOVA was performed; “***”: *p* < 0.001.

To entrap exosomes in ECM of host cells, according to the methods from previous researches (Alvarez-Erviti et al., 2011; Zhang et al., 2019), we fused a targeting peptide CBD with the extra-exosomal N-terminus of Lamp2b and overexpressed this fusion protein in host cells (Supplementary Figure S1). Since the heptapeptide CBD derived from collagenase can specifically bind to collagen I in ECM, we expect that the fused CBD in exosome membrane protein Lamp2b can lead to the entrapment of exosomes in ECM. The overexpression of CBD-Lamp2b fusion protein in HFF was confirmed by the results of qRT-PCR and Western blot analysis (Figures 2A–C).

To investigate whether modified exosomes could be entrapped and enriched into the ECM surrounding HFF cells, we first assessed and measured the number of exosomes surrounding the host HFF cells exhibited in morphological images acquired by SEM (Figure 2D). We found that more exosomes (extracellular vesicles with 40–150 nm of diameter) were attached on the surface of HFF-CBD-Lamp2b cells than them on surfaces of the HFF-Lamp2b and HFF cells (Figure 2E). In addition, we further examined the expression levels of exosomes markers (Hsp90, TSG101, and CD63) in the whole proteins of cells by Western blot. We found that the expression levels of Hsp90, TSG101, and CD63 were higher in HFF-CBD-Lamp2b cells than them in HFF cells and HFF-Lamp2b cells when the protein levels of ECM markers (fibronectin, collagen I, laminin, and aggrecan) and internal control of β-actin are almost same (Figure 2F and Supplementary Figure S2). The exosomal particle number measurement by NTA showed that there were less number of exosomes in the supernatant of HFF-CBD-Lamp2b cells than them in the supernatants of HFF cells and HFF-Lamp2b cells (Figure 2G). These results suggest that the CBD-Lamp2b fusion protein preferentially entraps the exosomes in the ECM around host cells.

### Preparation and Characterization of Exosomes Enriched in ECM

**FIGURE 3:**
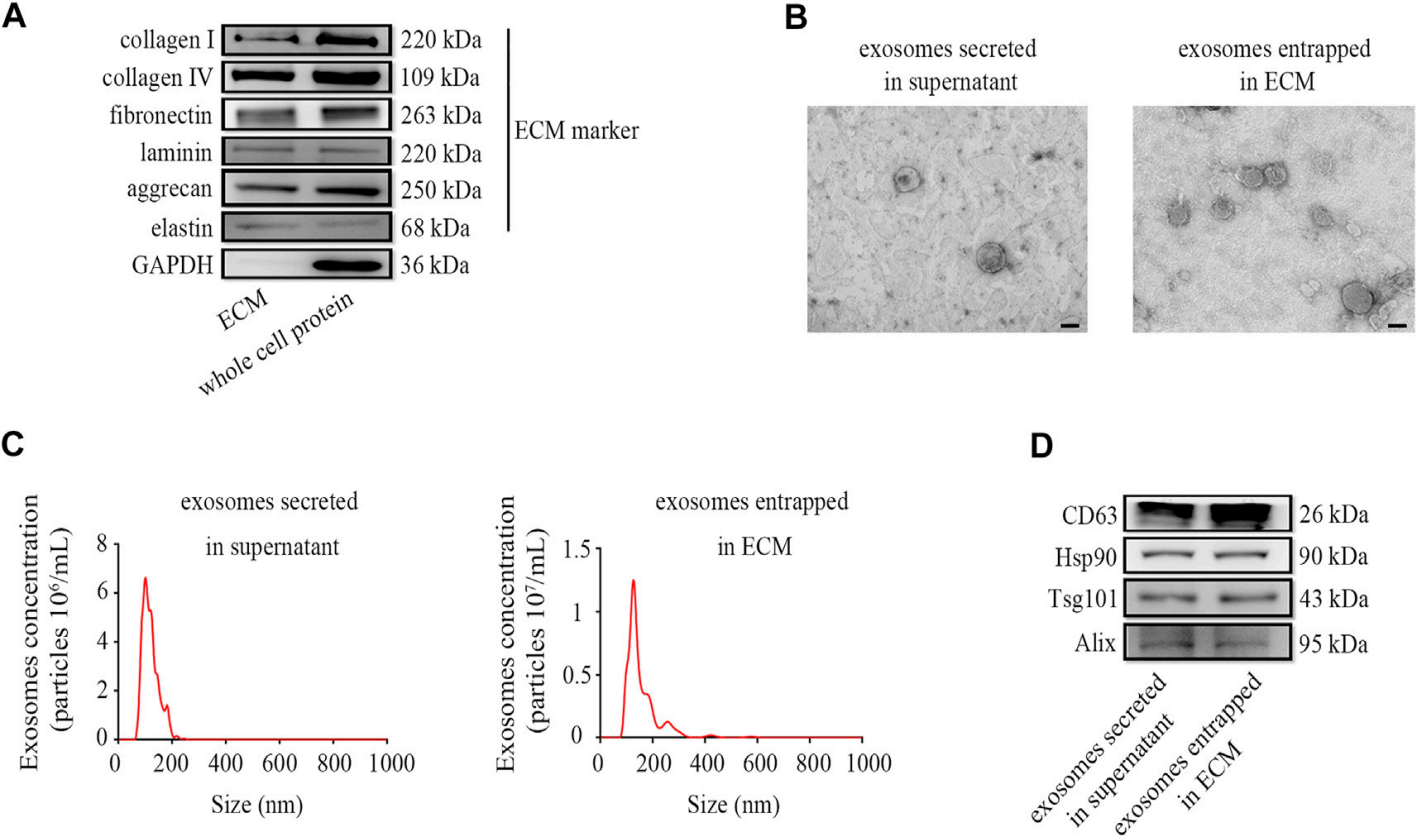
Preparation and characterization of exosomes enriched in ECM. **(A)** The markers of liquid ECM were determined by Western blot. **(B)** TEM images for exosome morphology. **(C)** NTA analysis of particle size, and **(D)** Western blot analysis for exosomal markers of exosomes entrapped in ECM from HFF-CBD-Lamp2b cells and those secreted in supernatant from HFF cells. Scale bar = 100 nm.

To verify that the ectopic expression of CBD-Lamp2b fusion protein can entrap the exosomes into the ECM of host cells and the exosomes can be enriched by liquid ECM preparation, we developed a repeated freeze-thaw approach to obtain the liquid ECM without destroying the membrane structure of exosomes (Lu et al., 2011, 2012; Fernández-Pérez and Ahearne, 2019; Li et al., 2019). The results from Western blot confirmed that the liquid ECM was successfully prepared by assessment of the ECM components such as collagen I, collagen IV, fibronectin, laminin, aggrecan and elastin from liquid ECM and whole cell protein (Figure 3A). Since the GAPDH is a conserved protein localized in cytoplasm, the lack of GAPDH expression in ECM indicated the success of decellularization (Figure 3A).

To verify the quality of exosomes in the liquid ECM, we characterized the exosomes entrapped in ECM from HFF-CBD-Lamp2b cells compared to those secreted in supernatant from HFF cells by the analysis of Western blot, TEM and NTA. The results from TEM image and NTA analysis showed that the particle of exosomes enriched in ECM has the size around 130 nm diameters that was almost the same as the size of exosomes secreted in medium. Exosomes in both ECM and medium had the similar biofilm structures that were consistent with those previously reported (Figures 3B,C) (Alvarez-Erviti et al., 2011; Wu et al., 2020). The results from Western blot confirmed the enrichment of exosomes in ECM by the detection of exosomal markers such as CD63, Hsp90, Tsg101, and Alix (Figure 3D).

**FIGURE 4:**
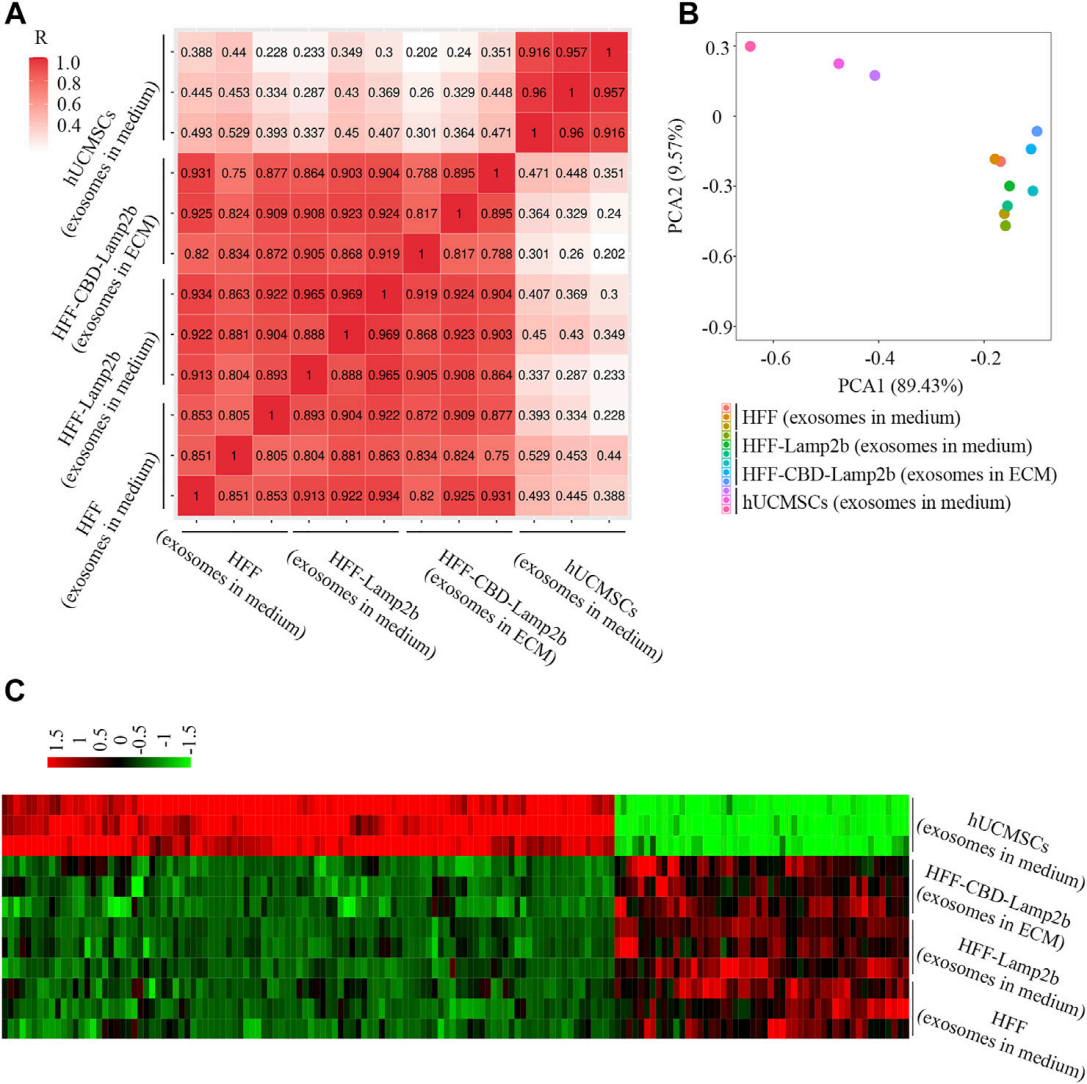
The comparison of exosomal miRNAs from ECM or media derived from HFF-CBD-Lamp2b cells, HFF cells, HFF-Lamp2b cells and hUCMSCs. **(A)** Spearman’s correlation analysis. **(B)** Principal component analysis was used to investigate the differences of exosomal miRNA from indicated samples. **(C)** The heatmap was used to show the expression of different miRNAs from indicated samples (red color means upregulation; green color means downregulation).

To further investigate the quality of exosomes enriched in ECM, we profiled the exosomal miRNAs using next-generation sequencing technology and compared the miRNAs in ECM exosomes from HFF-CBD-Lamp2b cells with those in medium exosomes from HFF cells, HFF-Lamp2b cells and hUCMSCs by spearman correlation analysis, principal component analysis (PCA) and heatmap analysis. The results from spearman correlation analysis showed the correlation coefficients between any two exosome samples from HFF cells medium, HFF-Lamp2b cells medium and from HFF-CBD-Lamp2b cells ECM had a high correlation (R > 0.75); however, the exosomes from HFF-CBD-Lamp2b cells ECM, from the media of HFF and HFF-Lamp2b had a low correlation (R < 0.5) with the exosomes from the medium of hUCMSCs (Figure 4A). The PCA showed that the distribution of exosomes from the media of HFF cells and HFF-Lamp2b cells, and the ECM of HFF-CBD-Lamp2b cells were different from the exosomes from hUCMSCs medium. The exosomes from hUCMSCs medium strayed far from the other three types of exosomes (Figure 4B). These results indicated that the miRNAs from these three types of exosomes were similar and high correlation. The results from heatmap analysis (Figure 4C) also demonstrated that the exosomes from the media of HFF and HFF-Lamp2b cells, and from the ECM of HFF-CBD-Lamp2b showed similar expression pattern compared to the exosomes from hUCMSCs medium. We found that the expression levels of 154 kinds of miRNAs from exosomes in the media of HFF and HFF-Lamp2b cells, and in the ECM of HFF-CBD-Lamp2b were significantly different compared with miRNAs levels from exosomes in hUCMSCs medium. Among the differentially expressed miRNAs, 104 miRNAs were up-regulated (fold change > 2) and 50 miRNAs were down-regulated (fold change < 0.5) in exosomes from hUCMSCs medium as compared to the other three exosomes (Figure 4C). The heatmap of miRNA expression showed high inter-group similarity for the exosomes from the media of HFF and HFF-Lamp2b cells, and the exosomes from ECM of HFF-CBD-Lamp2b. Overall, overexpression of CBD-Lamp2b will not change the quality of exosomes enriched in ECM compared to the exosomes in the media of HFF host cells.

### Mi29-Exo-ECM Biomaterial Inhibited TGF-β-Induced Accumulation of Collagen I *in Vitro*

**FIGURE 5:**
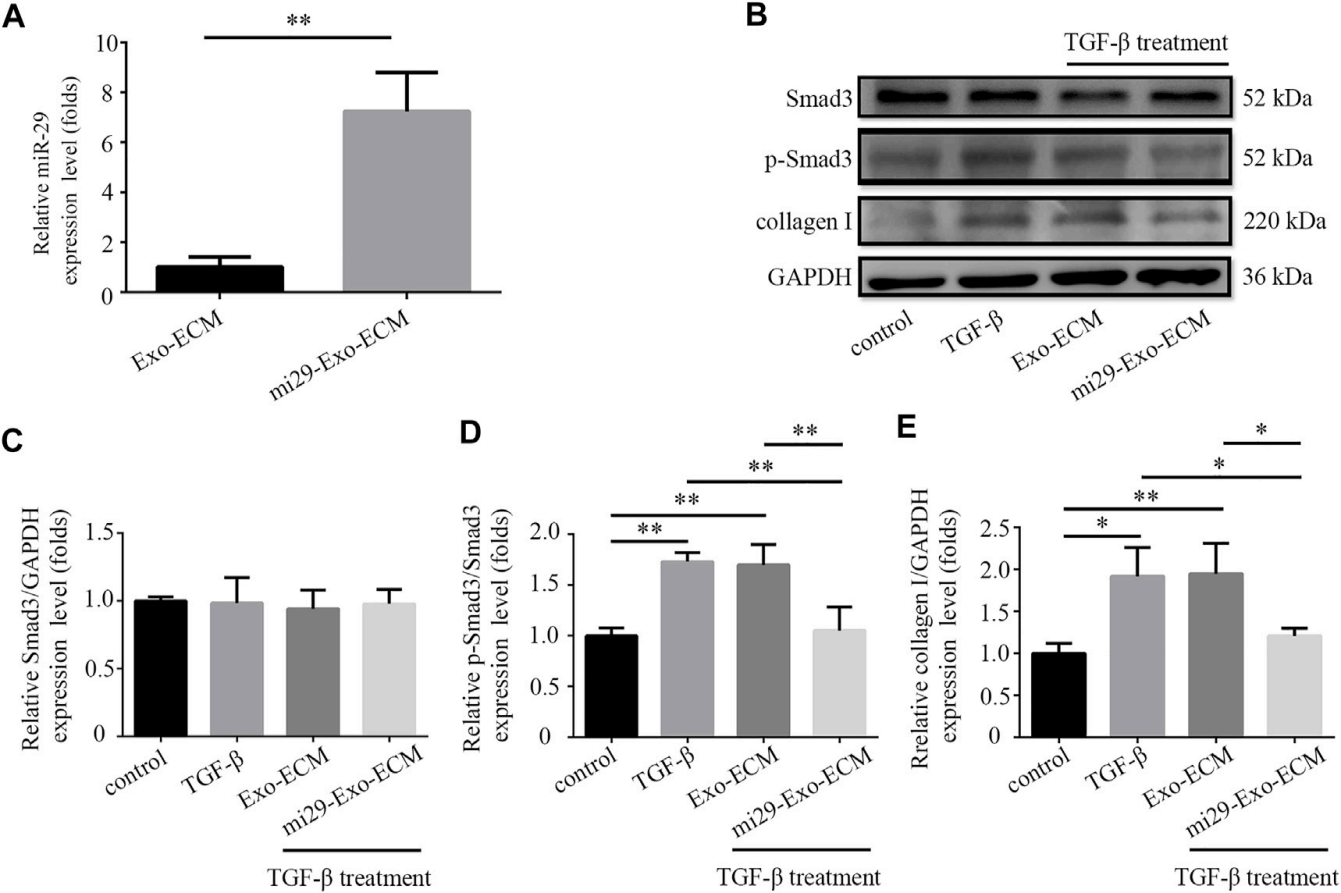
Mi29-Exo-ECM inhibited TGF-β-induced accumulation of collagen I in NIH 3T3 cells. **(A)** Expression level of miR-29 in mi29-Exo-ECM was determined by qRT-PCR. “Exo-ECM”: exosomes from ECM of HFF-CBD-Lamp2b cells; “mi29-Exo-ECM”: exosomes from mi29-Exo-ECM. Data are represented as mean ± SD (*n* = 3). Student’s t-test was performed; “**”: *p* < 0.01. **(B)** The changes of collagen I protein levels and phosphorylation levels of Smad3 were analyzed by Western blot. Relative expression levels of Smad3 **(C)**, *p*-Smad3/Smad3 **(D)**, collagen I **(E)** were quantified. The GAPDH was used as an internal control. “Control”: NIH 3T3 cells; “TGF-β”: TGF-β-induced NIH 3T3 cells; “Exo-ECM”: Exo-ECM treatment; “mi29-Exo-ECM”: mi29-Exo-ECM treatment; Data are represented as mean ± SD (*n* = 3). ANOVA was performed; “*”: *p* < 0.05; “**”: *p* < 0.01.

PF is associated with an excessive accumulation of collagens (He et al., 2013). It is well-known that the miR-29 is a major regulator of genes associated with PF (Xiao et al., 2012; Montgomery et al., 2014). Overexpression of miR-29 can negatively regulate TGF-β expression and Smad3 signaling activation that are correlated with the severity of the fibrosis (Cushing et al., 2011; He et al., 2013). Here, the miR-29 was overexpressed in HFF-CBD-Lamp2b cells to produce the liquid mi29-Exo-ECM. The result from qRT-PCR analysis (Figure 5A) showed that the expression levels of miR-29 in mi29-Exo-ECM were significantly higher than them in control Exo-ECM that was derived from HFF-CBD-Lamp2b cells without miR-29 ectopic expression. To evaluate whether mi29-Exo-ECM biomaterial can function on PF, we first investigate its function on reduction of collagen I, α-SMA (alpha-smooth muscle actin) expression and inactivation of Smad3 in TGF-β-induced NIH 3T3 cells. The TGF-β treatment clearly increased the expression of collagen I, α-SMA and phosphorylation of Smad3 (p-Smad3) in NIH 3T3 cells; only the mi29-Exo-ECM effectively reduced the levels of p-Smad3, collagens I and α-SMA in TGF-β-induced NIH 3T3 cells compared to the Exo-ECM (Figures 5B–E and Supplementary Figures S3A,B). Meanwhile, mi29-Exo-ECM could significantly down regulate the mRNA levels of Col1a1 and Col3a1 that were generally highly expressed in NIH 3T3 cells (Supplementary Figures S3C,D).

### Local Administration of Liquid mi29-Exo-ECM Efficiently Reduce PF *in Vivo*

**FIGURE 6:**
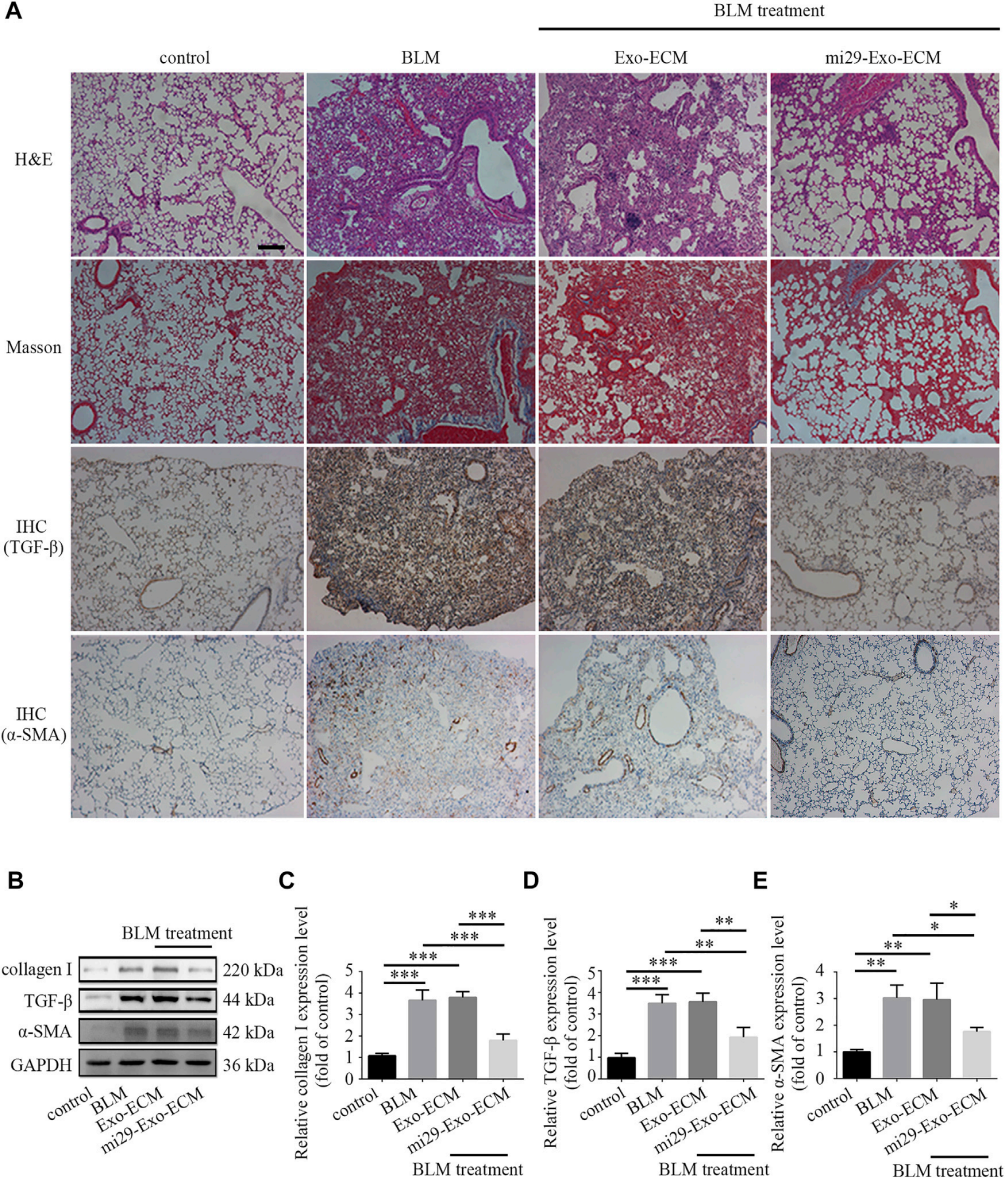
Pulmonary fibrosis therapeutic potential of mi29-Exo-ECM biomaterial. **(A)** H&E staining, Masson staining and IHC of lung tissues from mice treated with BLM, Exo-ECM biomaterial, mi29-Exo-ECM biomaterial. Scale bar = 1,000 μm. **(B)** The changes of collagen I, TGF-β and α-SMA protein levels in pulmonary fibrosis mice after treated with mi29-Exo-ECM biomaterial. Relative expression levels of collagen I **(C)**, TGF-β **(D)**, α-SMA **(E)** were quantified. “Control”: no treatment mice; “BLM”: bleomycin-induced pulmonary fibrosis mice model; “Exo-ECM”: Exo-ECM treatment; “mi29-Exo-ECM”: mi29-Exo-ECM treatment; Data are represented as mean ± SD (*n* = 5). ANOVA was performed; “*”: *p* < 0.05; “**”: *p* < 0.01; “***”: *p* < 0.001.

To confirm the therapeutic potential of mi29-Exo-ECM for PF *in vivo*, bleomycin-induced PF mice were intratracheally administered with total 600 μg liquid ECM with about 25 pmol exosomes with or without miR-29 enrichment, mi29-Exo-ECM or Exo-ECM. After 11 days of treatment, the mice were sacrificed and the lung tissues were harvested for H&E, Masson and IHC staining, and Western blot analysis. The upregulations of TGF-β and α-SMA are well known critical markers of tissue epithelial-mesenchymal transition (EMT) and PF (Cushing et al., 2011; Lemoinne et al., 2016). The results from H&E, Masson staining, and IHC analysis for TGF-β and α-SMA showed that the bleomycin treatment successfully caused the fibrosis and upregulation of TGF-β and α-SMA in lung lesion areas in mice, and mi29-Exo-ECM treatment dramatically restored lung textures from fibrosis and decreased the expression levels of TGF-β and α-SMA compared to Exo-ECM treatment (Figure 6A and Supplementary Figure S7). The Western blot assay further demonstrated that the mi29-Exo-ECM reduced the expression levels of collagen I, TGF-β and α-SMA in PF tissues of mice (Figures 6B–E). Treatment with mi29-Exo-ECM lowered the Ashcroft scores significantly and down-regulated the levels of HYP in lungs in bleomycin-induced PF mice (Supplementary Figures S5, S6). These results were consistent with those observed in the pathological sections, which suggested that the mi29-Exo-ECM treatment restored lung textures from fibrosis. Overall, the results from Figure 6; Supplementary Figures S5, S6 indicated that mi29-Exo-ECM showed the good repair effect on PF *in vivo* due to the enriched exosomes loaded with miR-29.

## Discussion

In this study, we successfully developed a type of functional ECM biomaterial enriched with miR-29-loaded exosomes and investigated the therapeutic effect of the biomaterials on PF. This biomaterial has some features as follows: 1) the liquid ECM biomaterial can entrap and enrich exosomes and provide an appropriate environment for exosomes to maintain the stability and to function; 2) as a carrier, exosomes can deliver small RNAs and protect them from degradation; 3) the overexpressed miR-29 can be recruited in and delivered by exosomes to function on the lesion of PF.

PF is a high mortality lung disease and no current treatment has proved effective to cure the disease. Many studies demonstrated that PF is associated with the abnormal activity of alveolar epithelial cells (AECs). These cells produce myofibroblast foci *via* stimulating the epithelial to mesenchymal transition (EMT) (Korfei et al., 2008; King Jr et al., 2011). In myofibroblast foci, the fibroblast secretes excessive amounts of ECM, mainly collagens, leading to scarring and destruction of the lung architecture (Gross and Hunninghake, 2001; King Jr et al., 2011; Martinez et al., 2017; Richeldi et al., 2017; Lederer and Martinez, 2018). Since TGF-β/Smad3 signaling is a critical factor for ECM gene expression, regulation of this signaling has been considered a potential approach for treatment of PF (Cushing et al., 2011; Xiao et al., 2012; He et al., 2013; Montgomery et al., 2014). For example, TGF-β antisense oligonucleotides has been delivered and targeted to lung to treat PF (Kim et al., 2020). In PF, TGF-β suppressed the level of miR-29 leading to the overexpression of collagens-related genes suggesting that miR-29 is an important mediator of TGF-β/Smad3 signaling. Moreover, it was reported that there was a complex feedback regulatory relationship between miR-29 and TGF-β/Smad3 signaling (Cushing et al., 2015). Sleeping beauty (SB)-mediated miR-29 gene transfection has proved effective to prevent and treat PF induced by bleomycin in mice (Xiao et al., 2012). Recent studies showed that miR-29 could regulate ECM production and deposition to alleviate PF treatment (Cushing et al., 2011; Cushing et al., 2015). According to the past studies, miR-29 has been proved to act as a “master fibro-miRNA” regulator to be involved in fibrosis (Deng et al., 2017). In PF, the down regulation of miR-29 level is negatively related to fibrosis and the expression level of profibrotic genes, such as Col1a1 and Col3a1 (Cushing et al., 2011; Xiao et al., 2012). MiR-29 plays a critical role in TGF-β-induced ECM production by activating the phosphatidyl inositol 3-kinase (*PI3K*)/protein kinase B (*AKT*) pathway and the Wnt/β-catenin pathway in human lung fibroblasts (Deng et al., 2017). Many previous studies showed that therapeutic delivery of miR-29 for PF decreased collagen expression and reversed PF (Cushing et al., 2011; Montgomery et al., 2014). MiR-29 is likely to become a new target in the diagnosis and evaluation of PF, as well as a new perspective and direction in which to search for new therapeutic targets of fibrosis.

It is well known that miRNAs have great potential for clinical therapy. However, direct administration of miRNAs in the body would face the problems of rapid diffusion and RNA enzyme degradation, which leads to shorter treatment duration. Therefore, package by liposomes, polymer nanoparticles, or virus can protect miRNAs from enzyme degradation and diffusion, and can prolong and enhance its function (Sercombe et al., 2015; Ha et al., 2016). Unlike typical nanoparticulate systems such as liposomes, polymeric nanoparticles or virus, exosomes are natural carriers for microRNA delivery and can deliver cargoes such as small RNAs directly into the cytoplasm potentially avoiding the endosomal pathway and lysosomal degradation, and therefore enhanced the transfection efficiency of small RNAs (Ha et al., 2016). For this great merit, exosomes have proved to offer a potential new paradigm in treatment of diseases.

Exosomes were secreted by most cells and distributed in different bodily fluids (e.g., blood, urine, and saliva) (Boriachek et al., 2018). However, how to isolate exosome remains an obstacle for it application. Existing methods for exosome isolation mainly rely on a large collection of medium and heavy ultracentrifugation. Although the ultracentrifugation-based methods were currently considered as the gold standard of exosome isolation, it had some disadvantages such as high equipment cost, time-consuming, large sample volume required, low recovery, and purity (Li P. et al., 2017). Other separation methods currently developed include sucrose-gradient centrifugation, coprecipitation, size-exclusion chromatography, and field flow fractionation. Their advantages have been reported, such as easy and user-friendly processing, high yield, wide variety of eluents and broad separation range. However, they also faced the problems of lengthy duration and fractionation equipment required (Xu et al., 2016; Coumans et al., 2017; Shao et al., 2018). To optimize exosome isolation, we attempted to develop a novel method to entrap and enrich the secreted exosomes to ECM of cell surface and to reduce their diffusion in the medium during long time cell culture. CBD is a heptapeptide (TKKTLRT) derived from collagenase and could specifically bind to collagen I, one of the main compounds in ECM (Jiang et al., 2019; Zhang et al., 2019). In our previous study, we overexpressed fusion proteins CBD-IDE and CBD-MME in HFF cells and developed a type of liquid ECM biomaterial enriched with CBD-IDE and CBD-MME relying on the binding of CBD and collagen I in ECM. This biomaterial successfully reduced the Aβ accumulation and Tau phosphorylation in AD cell models (Zhang et al., 2019). Lamp2b was a protein found abundantly in exosomal membranes (Alvarez-Erviti et al., 2011). By fusing CBD to the extra-exosomal N terminus of Lamp2b, the secreted exosomes were entrapped in ECM surrounding the host cells *via* the binding of CBD on external membrane of exosomes and collagen I in ECM. In this case, exosomes can be enriched in ECM and easily isolated by collecting the liquid ECM.

In this study, we enriched the miR-29 into exosomes and entrapped exosomes into ECM by overexpressing miR-29 and CBD-Lamp2b in HFF host cells, and produced the functional ECM biomaterial enriched with miR-29-loaded exosomes through the repeated freeze-thaw method without destroying the exosome membrane. Finally, this composite mi29-Exo-ECM was used for the repair of PF and showed a good treatment effect. This novel mi29-Exo-ECM biomaterial has the following merits: 1) miR-29 is a potential regulator for therapy of PF; 2) as a natural vehicle, exosome can prevent miRNA from enzymatic hydrolysis, facilitate the spread of RNA over long distance, and increase the duration of RNA function; 3) ECM plays a positive role in improving the disease microenvironment and contributes to the repair of tissue wound (Hou et al., 2016; Li J. et al., 2017; Zhang et al., 2019); 4) ECM provides appropriate environment for the collection and function of exosomes.

Although the liquid mi29-Exo-ECM had a good effect on the treatment of PF, there were still some problems that need to be further solved in the future, such as multiple dosing modalities and intervals to achieve optimal treatment outcomes, and the quantification of miR-29 in exosomes to facilitate drug administration consistency. In this study, to preserve the integrity of exosomes enriched in the ECM around HFF cells, the repeated freeze-thaw method was used to prepare the decellularized ECM. Our results confirmed the integrity and enrichment of exosomes in liquid ECM. However, the cell membrane components are hardly removed completely, which may affect the quality assessment of mi29-Exo-ECM. In this study, the miR-29 was enriched in exosomes by simply overexpressing miR-29 in the host cells. In the future, more efficient approaches should be developed for orientated enrichment by the specific binding between special peptide fused in inner membrane proteins of exosome and RNA fragment integrated in miR-29.

## Conclusion

In conclusion, our proof-of-concept study first demonstrated that exosomes could be enriched in ECM by overexpression of fusion gene CBD-Lamp2b in host cells. Based on this novel strategy, we developed a kind of functional biomaterial containing liquid natural ECM enriched with miR-29-loaded exosomes for PF treatment. The liquid ECM provides an appropriate microenvironment for exosomes function in the foci of damaged tissues, and the exosome is ideal natural carrier for miRNAs delivery for therapeutic purpose. This functional biomaterial provides a promising platform for gene therapy that could potentially be applied to treat different diseases by loading different functional miRNAs in exosomes that can be enriched in the liquid ECM.

## Data Availability

The original contributions presented in the study are publicly available. This data can be found here: https://www.ebi.ac.uk/arrayexpress/experiments/E-MTAB-10905/.
