# Supplementary material for: The Extracellular Matrix Enriched With Exosomes for the Treatment on Pulmonary Fibrosis in Mice
Source: Front Pharmacol. 2021 Dec 6;12:747223. doi: 10.3389/fphar.2021.747223 (PMC8685953; doi:10.3389/fphar.2021.747223)
Supplement: Supplementary file 1 [file DataSheet1.pdf]

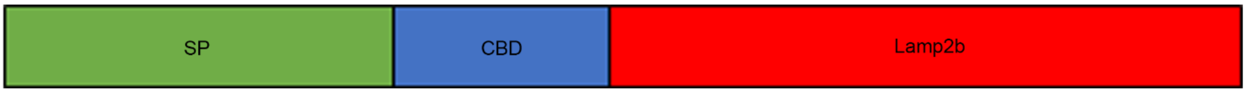

ATGGTGTGCTTCCGCCTCTTCCCGGTTCCGGGCTCAGGGCTCGTTCTGGTCTGCCT
AGTCCTGGGAGCTGTGCGGTCTTATGCAGGTAACTCGACTATGGGCAGTGGAACCA
AGAAGACCCTGAGAACCGGCAGTGGATCTGGATCCGGTGGCTCGAGTTTGGAACTT
AATTTGACAGATTCAGAAAATGCCACTTGCCTTTATGCAAAATGGCAGATGAATTTCAC
AGTTCGCTATGAAACTACAAATAAAACTTATAAAACTGTAACCATTTCAGACCATGGCA
CTGTGACATATAATGGAAGCATTTGTGGGGATGATCAGAATGGTCCCAAAATAGCAGT
GCAGTTCGGACCTGGCTTTTCCTGGATTGCGAATTTTACCAAGGCAGCATCTACTTAT
TCAATTGACAGCGTCTCATTTTCCTACAACACTGGTGATAACACAACATTTCCTGATG
CTGAAGATAAAGGAATTCTTACTGTTGATGAACTTTTGGCCATCAGAATTCCATTGAAT
GACCTTTTTAGATGCAATAGTTTATCAACTTTGGAAAAGAATGATGTTGTCCAACACTA
CTGGGATGTTCTTGTACAAGCTTTTGTCCAAAATGGCACAGTGAGCACAAATGAGTT
CCTGTGTGATAAAGACAAAACTTCAACAGTGGCACCCACCATACACACCACTGTGCC
ATCTCCTACTACAACACCTACTCCAAAGGAAAAACCAGAAGCTGGAACCTATTCAGTT
AATAATGGCAATGATACTTGCCTGCTGGCTACCATGGGGCTGCAGCTGAACATCACT
CAGGATAAGGTTGCTTCAGTTATTAACATCAACCCCAATACAACTCACTCCACAGGCA
GCTGCCGTTCTCACACTGCTCTACTTAGACTCAATAGCAGCACTATTAAGTATCTAGA
CTTTGTCTTTGCTGTGAAAAATGAAAACCGATTTTATCTGAAGGAAGTGAACATCAGC
ATGTATTTGGTTAATGGCTCCGTTTTCAGCATTGCAAATAACAATCTCAGCTACTGGGA
TGCCCCCCTGGGAAGTTCTTATATGTGCAACAAAGAGCAGACTGTTTCAGTGTCTGG
AGCATTTCAGATAAATACCTTTGATCTAAGGGTTCAGCCTTTCAATGTGACACAAGGA
AAGTATTCTACAGCCCAAGAGTGTTCGCTGGATGATGACACCATTCTAATCCCAATTAT
AGTTGGTGCTGGTCTTTCAGGCTTGATTATCGTTATAGTGATTGCTTACGTAATTGGCA
GAAGAAAAAGTTATGCTGGATATCAGACTCTGTAA

SUPPLEMENTARY FIGURE S1 The expression cassette of the modified Lamp2b protein. SP: signal peptide; CBD: collagen-binding domain; Lamp2b: lysosomal associated membrane protein 2b. (green color means SP; blue color means CBD; red color means Lamp2b).

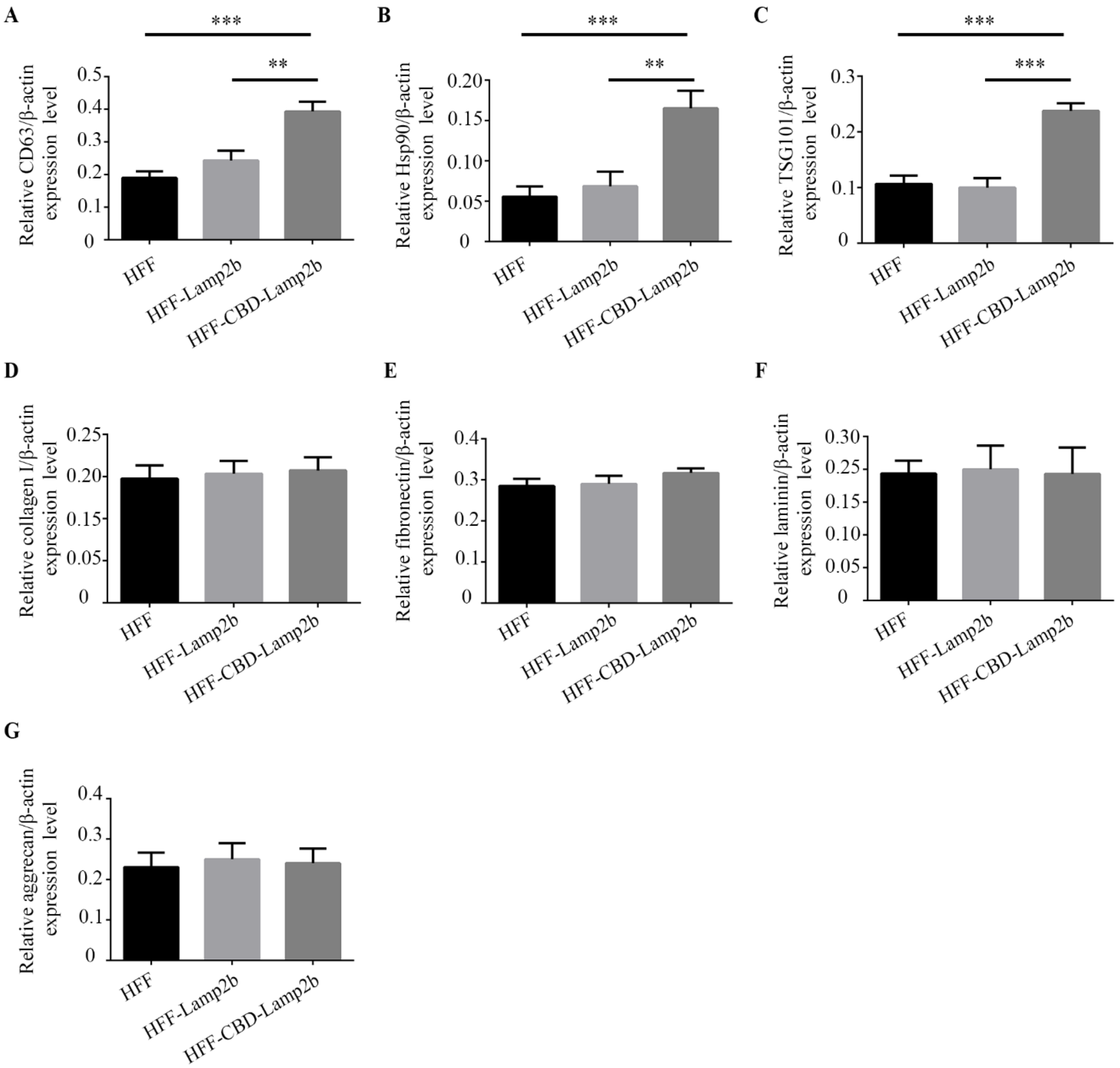

SUPPLEMENTARY FIGURE S2 Relative expression levels of CD63 **(A)**, Hsp90 **(B)**, TSG101 **(C)**, collagen I **(D)**, fibronectin **(E)**, laminin **(F)** and aggrecan **(G)** were quantified. The β-actin was used as an internal control. Data are represented as mean ± SD (n = 3). ANOVA was performed; "**" : $p < 0.01$; "***" : $p < 0.001$.

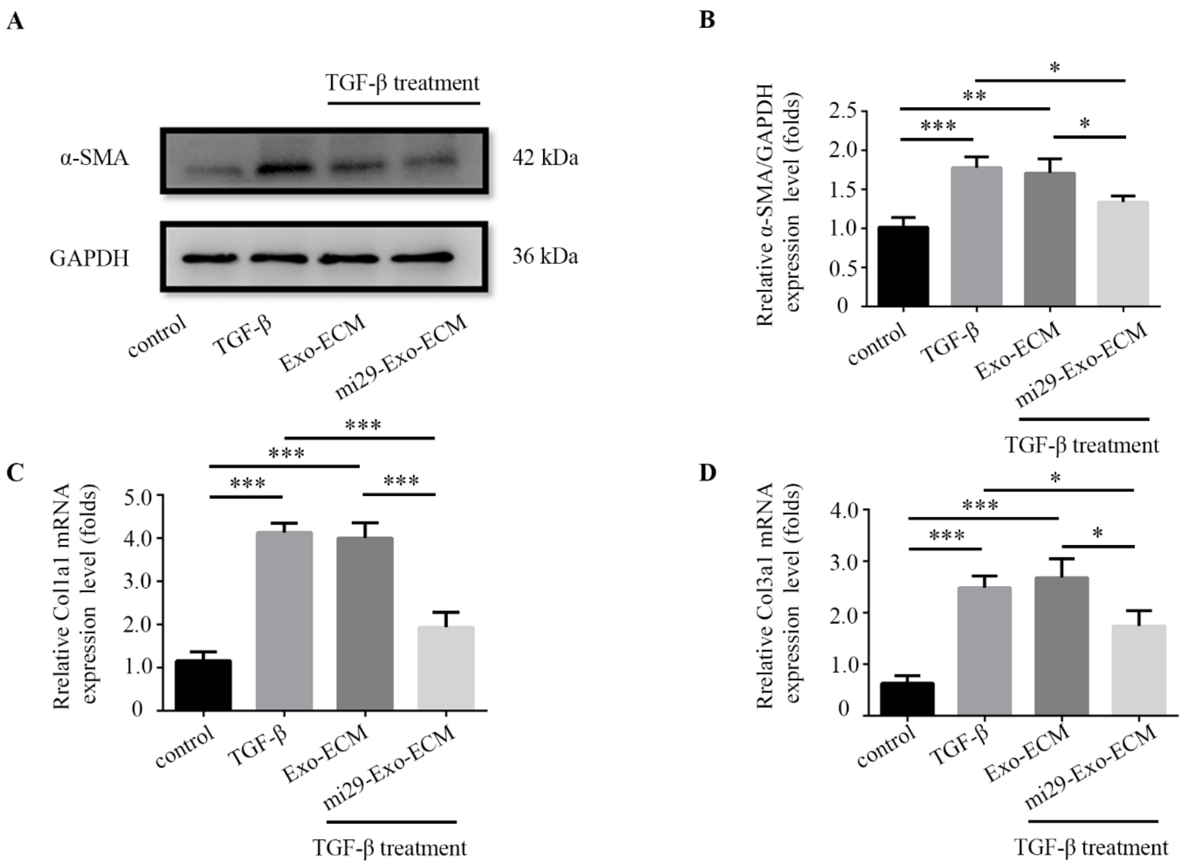

SUPPLEMENTARY FIGURE S3 The change of α-SMA protein level was analyzed by Western blot **(A)**. Relative expression level of α-SMA were quantified **(B)**. The GAPDH was used as an internal control. mRNA levels of Col1a1 **(C)** and Col3a1 **(D)** were determined by qRT-PCR. “control”: NIH 3T3 cells; “TGF-β”: TGF-β-induced NIH 3T3 cells; “Exo-ECM”: Exo-ECM treatment; “mi29-Exo-ECM”: mi29-Exo-ECM treatment; Data are represented as mean ± SD (n = 3). ANOVA was performed; “*” : $p < 0.05$; “**” : $p < 0.01$; “***” : $p < 0.001$.

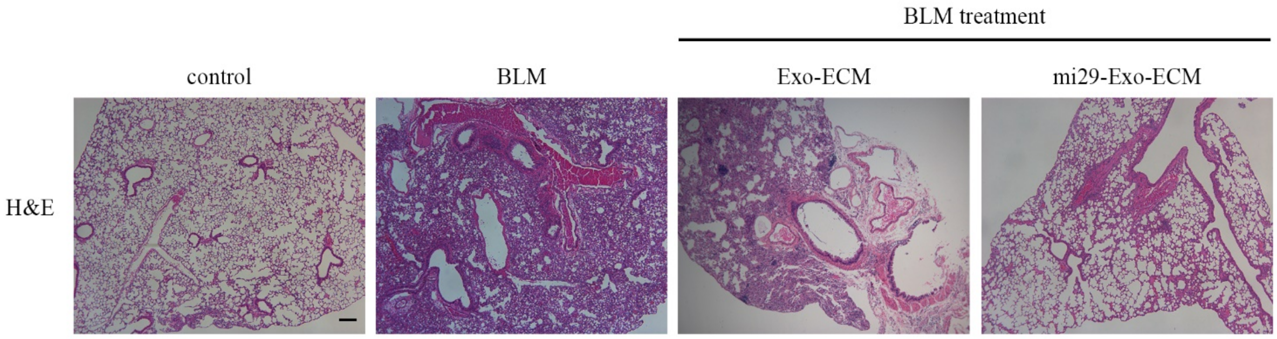

SUPPLEMENTARY FIGURE S4 The images of H&E staining with 40X magnification. Scale bar = 500 µm. “control”: no treatment mice; “BLM”: bleomycin-induced pulmonary fibrosis mice model; “Exo-ECM”: Exo-ECM treatment; “mi29-Exo-ECM”: mi29-Exo-ECM treatment.

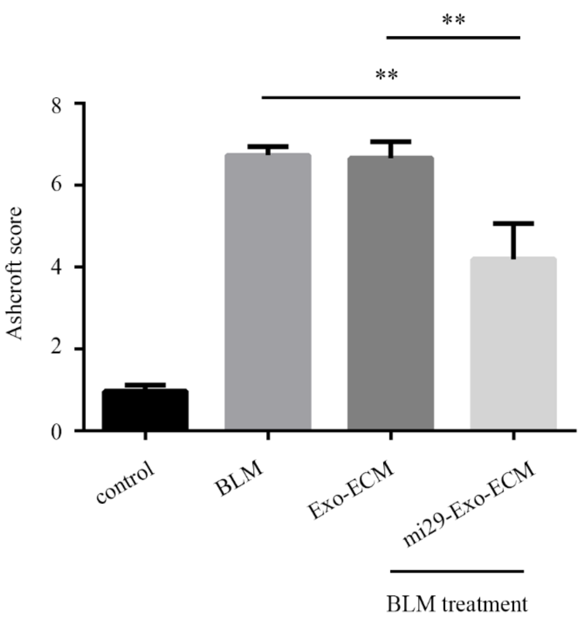

SUPPLEMENTARY FIGURE S5 The Ashcroft score of H&E staining from pulmonary fibrosis mice model. “control”: no treatment mice; “BLM”: bleomycin-induced pulmonary fibrosis mice model; “Exo-ECM”: Exo-ECM treatment; “mi29-Exo-ECM”: mi29-Exo-ECM treatment; Data are represented as mean ± SD (n = 5). ANOVA was performed; “**” : $p < 0.01$.

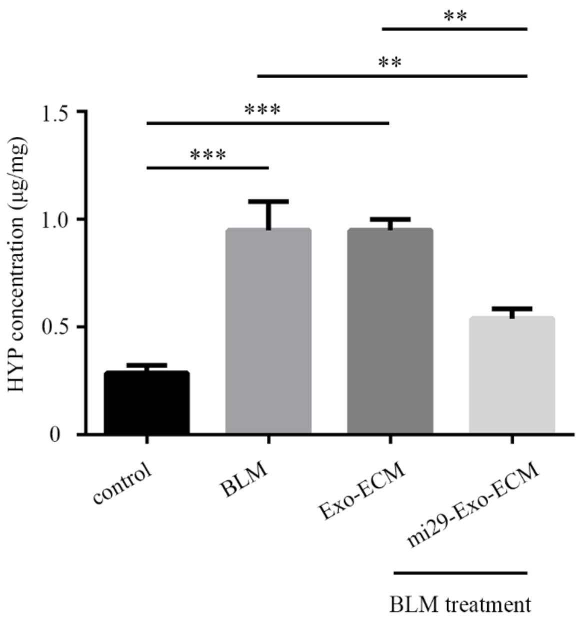

SUPPLEMENTARY FIGURE S6 The HYP concentration of lungs from pulmonary fibrosis mice model. “control”: no treatment mice; “BLM”: bleomycin-induced pulmonary fibrosis mice model; “Exo-ECM”: Exo-ECM treatment; “mi29-Exo-ECM”: mi29-Exo-ECM treatment; Data are represented as mean ± SD (n = 5). ANOVA was performed; “**” : $p < 0.01$; “***” : $p < 0.001$.

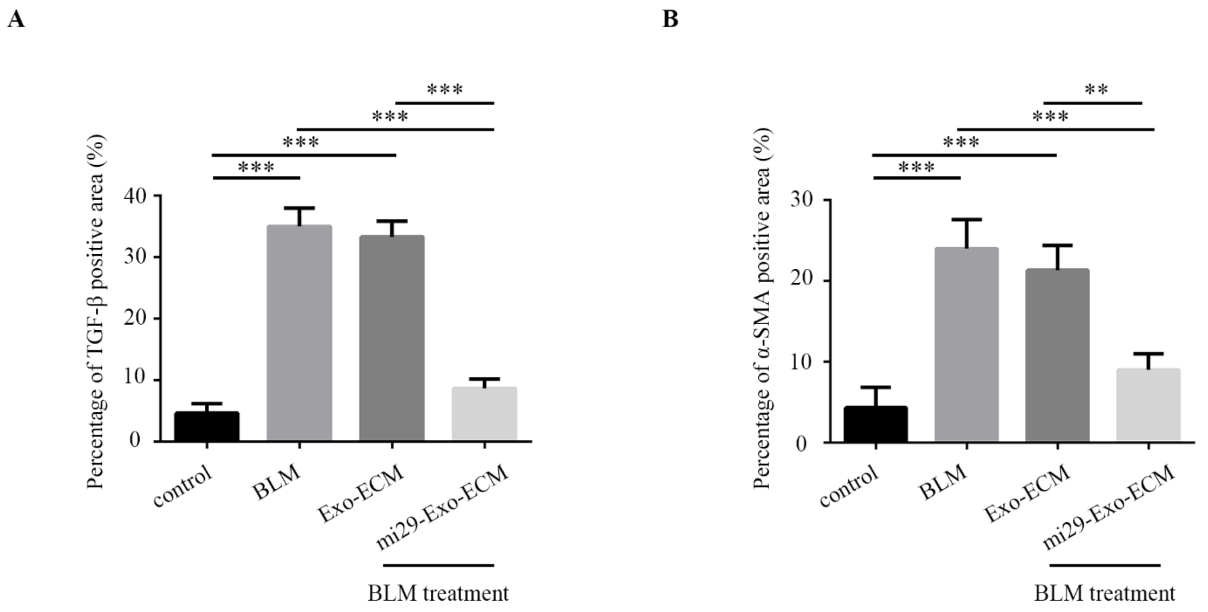

SUPPLEMENTARY FIGURE S7 The percentage of TGF-β **(A)** and α-SMA **(B)** positive area (%) from IHC. “control”: no treatment mice; “BLM”: bleomycin-induced pulmonary fibrosis mice model; “Exo-ECM”: Exo-ECM treatment; “mi29-Exo-ECM”: mi29-Exo-ECM treatment; Data are represented as mean ± SD (n = 5). ANOVA was performed; “**” : $p < 0.01$; “***” : $p < 0.001$.

SUPPLEMENTARY TABLE S1 The primers for PCR

Genes	Application	Primer Sequences
Lamp2b	cDNA clone	Fd: 5'-CCGACTAGTGCCACCATGGTGTGCTTCCGCCTCT-3'<br>Re:<br>5'-CGCGCGGCCGCTTACAGAGTCTGATATCCAGCATAACTT-3'
CBD-Lamp2b	cDNA clone	Fd: 5'-CCGACTAGTGCCACCATGGTGTGCTTCCGCCTCT-3'<br>Re:<br>5'-CGCGCGGCCGCTTACAGAGTCTGATATCCAGCATAACTT-3'<br>Fd:<br>5'-ACCAAGAAGACCCTGAGAACCGGCAGTGGATCTGGATCCG-3'<br>Re:<br>5'-GGTTCTCAGGGTCTTCTTGGTTCCACTGCCCATAGTCGAGTTAC-3'
miRNA-29	cDNA clone	Fd:<br>5'-CCGGTAACCGATTTCAAATGGTGCTACTCGAGTAGCACCA

		TTTGAAATCGGTTATTTTTG-3'
		Re:
		5'-AATTCAAAAAATAACCGATTTCAAATGGTGCTACTCGAGT AGCACCATTTGAAATCGGTTA-3'
Lamp2b	qRT-PCR	Fd: 5'-GCTGTGAAAAATGAAAACCGATT-3'
		Re: 5'-AGCTGAGATTGTTATTTGCAATGC-3'
GAPDH	qRT-PCR	Fd: 5'-ACCCACTCCTCCACCTTTGA-3'
		Re: 5'-TGTTGCTGTAGCCAAATTCGTT-3'
miRNA-29	qRT-PCR	Fd: 5'-CTGGTAGGTAGCACCATTTGAAAT-3'
		Re: 5'-AACTGGTGTCGTGGAGTCGG-3'
U6	qRT-PCR	Fd: 5'-CTCGCTTCGGCAGCACA-3'
		Re: 5'-AACGCTTCACGAATTTGCGT-3'
miRNA-29	RT-PCR	5'-CTCAACTGGTGTCGTGGAGTCGGCAATTCAGTTGAGTAA CCGAT-3'

Note: “Fd”: forward primer; “Re”: reverse primer
